# Advances in Neuroprotective Ingredients of Medicinal Herbs by Using Cellular and Animal Models of Parkinson's Disease

**DOI:** 10.1155/2013/957875

**Published:** 2013-09-01

**Authors:** Sandeep Vasant More, Hemant Kumar, Seong Mook Kang, Soo-Yeol Song, Kippeum Lee, Dong-Kug Choi

**Affiliations:** Department of Biotechnology, College of Biomedical and Health Science, Konkuk University, Chungju 380-701, Republic of Korea

## Abstract

Parkinson's disease (PD) is a multifactorial disorder, which is neuropathologically identified by age-dependent neurodegeneration of dopaminergic neurons in the substantia nigra. Development of symptomatic treatments has been partly successful for PD research, but there remain a number of inadequacies in therapeutic strategies for the disease. The pathogenesis of PD remains intricate, and the present anti-PD treatments appears to be clinically insufficient. Comprehensive research on discovery of novel drug candidates has demonstrated that natural products, such as medicinal herbs, plant extracts, and their secondary metabolites, have great potential as therapeutics with neuroprotective activity in PD. Recent preclinical studies suggest that a number of herbal medicines and their bioactive ingredients can be developed into optimum pharmaceuticals for treating PD. In many countries, traditional herbal medicines are used to prevent or treat neurodegenerative disorders, and some have been developed as nutraceuticals or functional foods. Here we focus on recent advances of the evidence-linked neuroprotective activity of bioactive ingredients of herbal origin in cellular and animal models of PD research.

## 1. Introduction

Parkinson's disease (PD) is a chronic neurological disorder, characterized by a selective loss of dopaminergic neurons in the substantia nigra (SN) of ventral midbrain area, causing a subsequent reduction of dopamine (DA) levels in the striatum. Loss of dopaminergic supply to striatum causes imbalance with neurotransmitters like acetylcholine and DA, resulting in PD symptoms. Some typical characteristic symptoms observed in PD patients are tremor, myotonia, and dyskinesia [1]. The three main strategic developments in drug discovery that have advanced the progress in therapeutic management of PD patients have focused on the alleviation of motor symptoms by the use of dopaminergic mimetics, the development of novel nondopaminergic drugs for symptomatic improvement, and lastly, the discovery of neuroprotective compounds that have disease modifying effects in PD [2]. The pathogenesis and etiology of PD are not completely understood. Extensive study of various models mimicking key features of PD has outlined important cellular factors of dopaminergic cell death, including neuroinflammation, oxidative stress, mitochondrial dysfunction, and excitotoxicity [3, 4]. Although no model has thus far been able to reiterate all the pathological features of PD [5], the neurotoxic models have proved themselves to be a worthy tool for developing novel therapeutic strategies and assessing the efficacy and adverse effects of symptomatic treatments of PD [6].

Since ancient times, PD has been documented in various parts of the world. Based on their experience-based theories as well as practices from elsewhere, Asian countries, such as India, China, Japan, and Korea, have been using different combinations of herbal materials to treat PD within the context of ancient herbal medical systems [7]. Ayurveda, an ancient form of alternative traditional medicine followed in the Indian subcontinent describes PD as “Kampavata” [8] wherein seed preparations of mucuna are used as contemporary medicine for the treatment of PD [7]. Upon scientific investigations, it was found that *Mucuna pruriens* contains levodopa, which provides long-term amelioration of Parkinsonism [9, 10]. Formulation of powdered seed of *Mucuna pruriens* also showed positive effects on PD patients in clinical trials, with quick onset of action and without concomitant increase in dyskinesia [11]. Zandopa (HP-200), a commercial preparation of *Mucuna pruriens*, is also available for the treatment of PD [12]. In Chinese traditional medicine, 22,500 medicinal herbs are in use throughout China, of which only a few have been successfully investigated in animal experiments or clinical trials for potential development into herbal formulations for treating PD [13].

The past decade has substantiated considerable interest in phytochemical bioactive constituents from herbal medicines, which can have long-term medicinal or health-promoting qualities in PD [14]. In comparison, many medicinal plants exhibit specific medicinal actions without serving a nutritional role in the human diet and may be used in response to specific health problems over short- or long-term intervals [15, 16]. Therefore, a scientific re-examination of these therapies in preclinical models is valuable for the development of novel neuroprotective drugs for PD [17]. According to estimates from the World Health Organization, by 2040, neurodegenerative diseases will exceed cancer as the principal cause of death in industrialized countries. Irrespective of our advances in understanding the pathogenesis of PD, pharmacological treatments by conventional medicine have not transpired into satisfactory results. Therefore, it is plausible that the use of bioactive compounds from natural sources may yield more appropriate potential candidates for the preventive treatment of PD [18].

Comprehensive research on the discovery of novel neuroprotective drug candidates has proven that natural products, such as plant extracts and their bioactive compounds, can have tremendous potential as lead neuroprotective candidates in PD treatment. To list a few compounds from herbal origin, apomorphine, rivastigmine, and PYM-50028 are under clinical investigation to be used as potential neuroprotective agents in PD [19]. Here, we have focused on recent advances in the research of herbal medicines and their bioactive ingredients used in animal and cellular neurotoxic models of PD, so as to facilitate future basic and clinical investigations.

## 2. Neuroprotective Activity of Bioactive Compounds from Herbal Medicines

### 2.1. Ginsenoside Rg1

Ginseng is the dried root and rhizome of *Panax ginseng* and *Panax notoginseng* (Araliaceae) [13]. Ginseng is a valuable herb in traditional medicine, which has been utilized for over many centuries, based on the theory that it is a general tonic for the promotion of vitality, health, and longevity. The aqueous extract of ginseng has been used to treat many kinds of disease including ischemia, anemia, diabetes mellitus, gastritis, and insomnia [20]. There are over 30 ginsenosides among which the main active ingredients responsible for its vivid pharmaceutical actions are ginsenoside Rb1, Rd, Re, and Rg1 [21].

**Figure 1:**
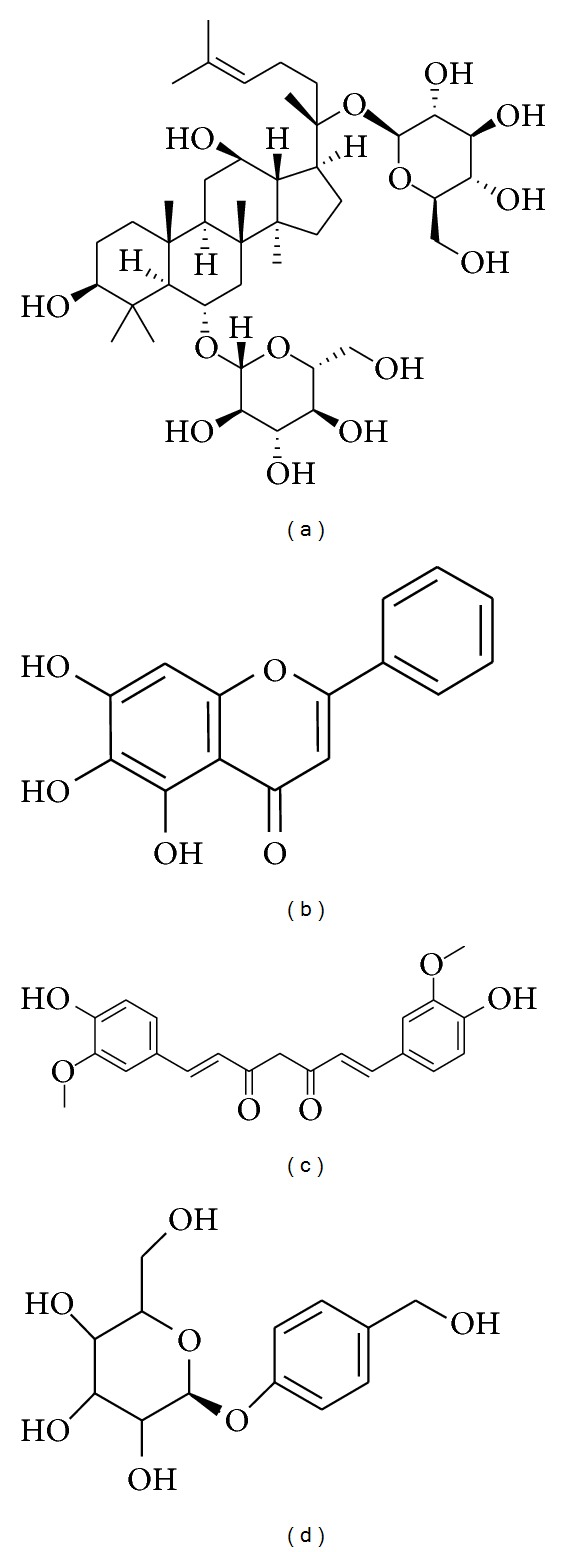
Chemical structure of ginsenoside Rg1 (a), baicalein (b), curcumin (c), and gastrodin (d).

Recently, the aqueous extract of *Panax ginseng* was investigated for its protective effects against cellular model of parkinsonism like 1-methyl-4-phenylpyridine (MPP^+^)-induced cytotoxicity in SH-SY5Y human neuroblastoma cells. In this study, the aqueous extract of *Panax ginseng* decreased the overproduction of reactive oxygen species (ROS), release of cytochrome c and activation of caspase-3, elevated Bax/Bcl-2 ratio, and thus, increased cell survival in MPP^+^-treated SH-SY5Y cells [20]. Apart from *Panax ginseng*, saponins, obtained from *Panax notoginseng* by the induction of thioredoxin-1, elicit a very potent neuroprotective effect on MPP^+^ induced toxicity to PC12 cells and Kunming mice [22, 23]. In a very recent report, ginsenoside Rg1 (Figure 1(a)) was studied for the mechanistic activity behind its antioxidant effect on hydrogen peroxide (H_2_O_2_)-induced oxidative stress to PC12 cells. Pretreatment with Rg1 at concentrations of 0.1–10 *μ*M significantly decreases the cytotoxicity induced by 400 *μ*M of H_2_O_2_ in PC12 cells. Ginsenoside Rg1 abates the phosphorylation and nuclear translocation of nuclear factor-kappa B (NF-*κ*B)/p65, phosphorylation, and degradation of inhibitor protein of *κ*B (I*κ*B), as well as the phosphorylation of I*κ*B-kinase complex (IKK). Furthermore, Rg1 also inhibited the activation of Akt and the extracellular signal-regulated kinase 1/2 (ERK1/2). These results indicate that ginsenoside Rg1 protects the cell injury induced by H_2_O_2_ via downregulating ERK1/2 and by decreasing the activation of the NF-*κ*B signaling pathway [24].

In a report by Xu et al., it was observed that pretreatment with ginsenoside Rg1 to MES23.5 cells renews an iron-induced reduction in mitochondrial transmembrane potential. Pretreatment with ginsenoside Rg1 also decreases the increase of iron influx by inhibiting 6-hydroxydopamine (6-OHDA)-induced upregulation of an iron importer protein divalent metal transporter 1 with iron responsive element (DMT1-IRE). Further findings demonstrated that, due to the antioxidant effect of ginsenoside Rg1, it inhibits iron regulatory proteins, and thereby downregulating DMT1 and IRE expression [25]. In a parallel study, pretreatment with ginsenoside Rg1 was seen to inhibit the MPP^+^-induced upregulation of DMT1-IRE, which was associated with the production of ROS and translocation of NF-*κ*B to nuclei in MES23.5 cells [26]. Similar results were reported in a 1-methyl-4-phenyl-1,2,3,6-tetrahydropyridine (MPTP) model of PD in C57BL/6 mice, wherein pretreatment with ginsenoside Rg1 significantly attenuated MPTP-induced elevated iron levels decreased the expression of DMT1 and increased ferroportin-1 expression in the SN [27]. Antiinflammatory effects of ginsenoside Rg1 were also evident in lipopolysaccharide (LPS)-induced microglial activation in male C57BL/6 mice. In this study, ginsenoside Rg1 is found to inhibit proinflammatory markers, including inducible nitric oxide synthase (iNOS), nitric oxide (NO), tumor necrosis factor alpha (TNF-*α*), and expression of ionized calcium binding adaptor molecule 1 (Iba-1) in both the cerebral cortex and hippocampus of C57BL/6 mice. Treatment with ginsenoside Rg1 suppresses downstream inflammatory markers by inhibiting the phosphorylation levels of I*κ*B, nuclear translocation of p65 subunit of NF-*κ*B, and phosphorylation level of ERK1/2 kinase induced by LPS [28]. Ginsenoside Rg1 is also reported to have protective effects on dopaminergic neurons in ovariectomized female SD rat injected intracerebroventricularly with 6-OHDA [29]. In a similar 6-OHDA-induced nigrostriatal injury model of PD, ginsenoside Rg1 was observed to have a neuroprotective effects on dopaminergic neurons through the insulin-like growth factor-I receptor signaling pathway [30].

### 2.2. Baicalein

Baicalein (Figure 1(b)) is a flavonoid and one of the active constituents obtained from a dried root of *Scutellaria baicalensis* (Labiatae). Recently, ethanolic extract of *Scutellaria baicalensis* was demonstrated to decrease LPS-induced expression of iNOS, NO, cyclooxygenase-2 (COX-2), and prostaglandin E_2_ levels in BV-2 and RAW 264.7 cells [31]. In a latest study by Li et al., they investigated the effects of baicalein on rotenone-induced neurotoxicity in PC12 cells. The results demonstrated that baicalein, in a concentration-dependent manner, inhibits the accumulation of ROS, deficiency of ATP, dissipation of mitochondrial membrane potential, and activation of caspase-3/7. Baicalein suppresses rotenone-induced apoptosis, indicating that baicalein likely improves mitochondrial function. Moreover, isolated rat brain mitochondria were used by the author to evaluate the effect of baicalein. It was found that treatment with baicalein promotes mitochondrial active respiration and prevents the rotenone-induced production of ROS, deficiency of ATP, and swelling of isolated brain mitochondria [32]. In addition to the rotenone model, baicalein was also explored for its neuroprotective effect in 6-OHDA-induced cellular and animal models of experimental parkinsonism. Baicalein at 0.5 and 5 *μ*g/mL promotes neurite outgrowth in PC12 cells and significantly attenuates the 6-OHDA-induced cell apoptosis in SH-SY5Y cells. In animal experiments, treatment with baicalein significantly attenuates muscle tremor in 6-OHDA-lesioned rats but does not have any effect on apomorphine induced rotations. Furthermore, baicalein treatment mitigates astroglial response and increases tyrosine-hydroxylase-(TH-) positive neurons in SN [33].

Analogous to this study, treatment with baicalein at 100, 200, and 400 mg/kg significantly attenuates muscle tremor in 6-OHDA-lesioned rats. Baicalein was demonstrated to modulate the balance between glutamate and gamma amino butyric acid. Baicalein was also demonstrated to inhibit cytochrome oxidase subunit I (CO-I) mRNA expression in the subthalamic nucleus [34]. In a similar study, baicalein was seen to improve impaired spontaneous motor activity and rotarod performance induced by MPTP in C57BL/6 mice. Besides, baicalein at 280 and 560 mg/kg displays a protective effect against the MPTP-induced fall of TH-positive neurons in the SN. Treatment with baicalein also abates an MPTP-induced decrease in DA levels in the striatum by changing dopamine catabolism and inhibiting dopamine turnover [35]. In a similar model of MPTP-induced loss of dopaminergic fibers in mice, pretreatment with baicalein was found to increase the levels of DA and 5-hydroxytryptamine in the striatum, increase the counts of dopaminergic neurons, and inhibit both the oxidative stress and the astroglial response [36].

Baicalein is also reported to decrease fibrillization of E46K and E46K *α*-synuclein-(*α*-syn-) induced aggregation and toxicity in N2A cells. It was also demonstrated that baicalein significantly attenuates both E46K-induced mitochondrial depolarization, significantly attenuates the inhibition of proteasome, and protects N2A cells against E46K-induced toxicity [37]. In a related study, baicalein was found to inhibit the oligomerisation of *α*-syn in cell-free and cellular systems, as well to act as an efficient inhibitor of *α*-syn fibrillation in cell-free systems. Furthermore, baicalein was demonstrated to inhibit the formation of *α*-syn oligomers in Hela and SH-SY5Y cells and protect SH-SY5Y cells from *α*-syn oligomer-induced toxicity [38].

### 2.3. Curcumin

Rhizomes of *Curcuma longa* (Zingiberaceae) with the common name of turmeric along with its active components have been comprehensively used in the Indian subcontinent as food additives and cosmetics, exhibiting several medicinal properties [39]. The multiple pharmacological activities of *Curcuma longa *are mainly attributed to its polyphenolic fraction, curcuminoids, comprised of curcumin (Figure 1(c)), demethoxy curcumin (DMC), and bis-demethoxy curcumin (BDMC). Following extensive research on curcumin, the major active component of curcuminoids has revealed its bioactivities, including antiinflammatory, antioxidant, proapoptotic, chemopreventive, chemotherapeutic, antiproliferative, wound healing, antinociceptive, antiparasitic, and antimalarial properties [40]. In a latest study by Jiang and coworkers, curcumin was found to ameliorate A53T *α*-syn-induced SH-SY5Y cell death by downregulating rapamycin/p70 ribosomal protein S6 kinase signaling [41]. In a similar study by Wang et al., curcumin was observed to decrease *α*-syn-induced intracellular ROS generation and inhibit caspase-3 activation in SH-SY5Y cells [42]. In a recent experiment by Ojha et al., they investigated curcuminoids for their neuroprotective effects on inflammation-mediated neurodegeneration of dopaminergic neurons of C57BL/6 mice in the acute MPTP-model. Authors found that oral pretreatment with curcuminoids (150 mg/kg/day) significantly prevents MPTP mediated loss of TH-positive neurons and depletion of DA. Furthermore, pretreatment with curcuminoids mitigates cytokines, generation of total nitrite, and the expression of protein inflammatory markers, such as glial fibrillary acidic protein (GFAP) and iNOS, in the striatum of MPTP-intoxicated mice. Moreover, curcuminoids also improved motor deficits produced by MPTP, as evidenced by rotarod and open field tests [43].

In a comparable study carried out by Pan et al., curcumin was observed to protect dopaminergic neurons from apoptosis in an MPTP mouse model of PD. Curcumin markedly ameliorated the loss of dopaminergic axons in the striatum as well as the demise of dopaminergic neurons, in an MPTP mouse model. Further mechanistic studies demonstrated that curcumin inhibits MPTP-induced hyperphosphorylation of c-Jun N-terminal kinase (JNK). Phosphorylation of JNKs is known to cause translocation of Bax to mitochondria as well as the release of cytochrome c, which ultimately results in mitochondria-mediated apoptosis. Authors have established that curcumin prevents the degeneration of nigrostriatal neurons by inhibiting the dysfunction of mitochondria through abolishing the hyperphosphorylation of JNKs induced by MPTP [44]. Apart from MPTP model, curcumin is also reported to be neuroprotective in a 6-OHDA-induced hemiparkinsonian mice model. Posttreatment with curcumin following a unilateral intrastriatal 6-OHDA injection to mice was found to decrease the 6-OHDA-induced loss of striatal TH fibers and nigral TH-immunoreactive neurons. The neuroprotection was accompanied with a significant weakening of astroglial and microglial reaction in the striatum and the substantia nigra pars compacta (SNpc). These results indicate that the neuroprotective effects of curcumin in 6-OHDA-lesioned mice may be mediated via its antiinflammatory properties, or direct protection on nigral DA neurons [45].

### 2.4. Gastrodin

*Gastrodia elata* (GE), belonging to the family of Orchidaceae, has been traditionally used as a folk medicine in Oriental countries for many centuries due to its vivid exhibition of therapeutic benefits [46]. The major compounds in GE are gastrodin, vanillyl alcohol, 4-hydroxybenzaldehyde, and vanillin (Figure 1(d)). These compounds are known to cross the blood brain barrier and also to display various biological activities, such as antioxidant, antiasthmatic, antimicrobial, and antimutagenic activities [47]. In a study by An et al., pretreatment with GE extract (10, 100, 200 *μ*g/mL) for 4 h prior to the addition of MPP^+^ significantly rescued the MPP^+^-induced decrease in viability of SH-SY5Y cells. Pretreatment with GE at 10, 100, and 200 *μ*g/mL for 4 h prior to the addition of 0.5 mM MPP^+^ significantly improves cell viability in Neuro-2a cells [46]. Pretreatment with GE (10, 100, and 200 *μ*g/mL) reduces the proportion of apoptotic cells, ROS, and Bax/Bcl-2 ratio in a concentration-dependent manner in MPP^+^-induced toxicity to SH-SY5Y cells [46]. These findings suggest that treatment with GE shifts the balance between pro- and antiapoptotic members towards cell survival.

Application of vanillyl alcohol to MPP^+^ intoxicated MN9D dopaminergic cells effectively improves cell viability and inhibits cytotoxicity. The underlying mechanisms of vanillyl alcohol were found to be attenuation of the elevated ROS levels, as well as initiating a decrease in the Bax/Bcl-2 ratio and poly (ADP-ribose) polymerase (PARP) proteolysis. These results demonstrate that vanillyl alcohol protects dopaminergic MN9D cells against MPP^+^-induced apoptosis by relieving oxidative stress and modulating the apoptotic process [47]. In a recent study, treatment with gastrodin significantly and dose dependently protected dopaminergic neurons against neurotoxicity, through regulating free radicals, Bax/Bcl-2 mRNA, and caspase-3 and cleaved PARP in SH-SY5Y cells stressed with MPP^+^ [48]. Gastrodin also shows neuroprotective effects in the subchronic MPTP mouse PD model by ameliorating bradykinesia and motor impairment in the pole and rotarod tests, respectively [48]. Consistent with this finding, gastrodin prevents DA depletion and reduces reactive astrogliosis caused by MPTP in SN and striatum of C57BL/6 mice. Moreover, gastrodin is also effective in preventing neuronal apoptosis by attenuating oxidative stress and apoptosis in SN and striatum of C57BL/6 mice. Gastrodin is also reported to significantly inhibit levels of neurotoxic proinflammatory mediators and cytokines including iNOS, COX-2, TNF-*α*, and IL-1*β* by inhibiting the NF-*κ*B signaling pathway and phosphorylation of MAPKs in LPS-stimulated microglial cells [49]. These results indicate that gastrodin has protective effects in experimental PD models and might be suitable for development as a clinical candidate to ameliorate PD symptoms [48].

### 2.5. Resveratrol

**Figure 2:**
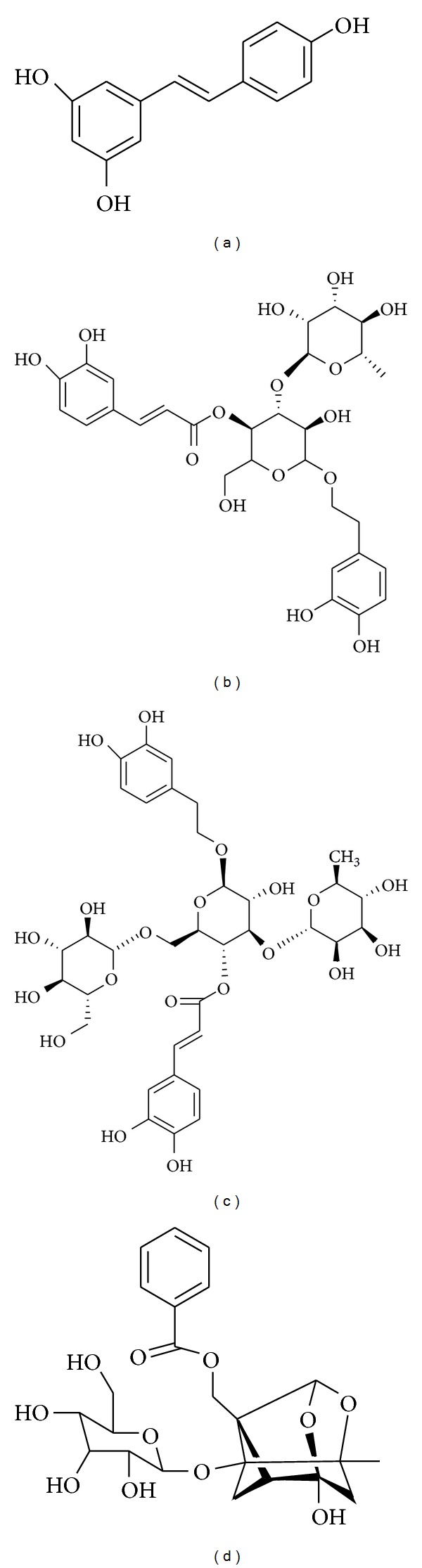
Chemical structure of resveratrol (a), acteoside (b), echinacoside (c), and paeoniflorin (d).

Resveratrol (Figure 2(a)) is a naturally occurring polyphenolic phytoalexin which occurs in plants such as grapes, peanuts, berries, and pines [50]. Resveratrol is reported to have several pharmacological properties, such as cardioprotection, scavenging of free radicals, and inhibition of COX and hydroperoxidase [50, 51]. In a recent study by Chang et al., resveratrol was found to markedly reduce levels of myeloperoxidase (MPO) in microglia and astrocytes, without increasing the levels of NO. Resveratrol-induced downregulation of MPO significantly attenuates rotenone-triggered inflammatory responses, including the production of ROS and phagocytic activity in primary microglia and astrocytes [52]. In addition, pretreatment with resveratrol also alleviates impaired responses to rotenone from primary mixed glia in MPO deficient mice. The authors further demonstrated that resveratrol attenuates rotenone-induced dopaminergic cell death in neuron-glia cocultures, as compared to *perse* neuronal culture. Similar effects were also shown by resveratrol in modulating MPO levels in microglia treated with MPP^+^, which supports its antiinflammatory profile in PD [52]. In an adjacent study by Wu et al., resveratrol was observed to protect SH-SY5Y cells against rotenone-induced apoptosis, and to enhance the degradation of *α*-syn in *α*-syn-expressing PC12 cell line via the induction of autophagy. After observing that suppression of silent information regulator 2 (SIRT1) and metabolic energy sensor AMP-activated protein kinase (AMPK) causes a decrease in protein levels of LC3-phosphatidylethanolamine conjugate (LC3-II), the authors concluded that AMPK and/or SIRT1 are required for the resveratrol-mediated induction of autophagy [53]. A similar study of the PC12 cell line demonstrated that pretreatment with resveratrol for 3 h before MPP^+^ significantly reduced apoptosis-mediated neuronal cell death [54]. The authors also established that resveratrol tunes mRNA levels and protein expression of Bax and Bcl-2. Further investigation revealed that resveratrol reduces apoptotic neuronal cell death by decreasing cytochrome c and nuclear translocation of the apoptosis-inducing factor (AIF) [54]. As compared to earlier neuroprotective mechanism reported for resveratrol, it was found that antiapoptotic effects elicited against MPP^+^ in rat cerebellar granule neurons by resveratrol are independent of the stimulation of mammalian SIRT-2, but dependent on its antioxidant properties [55].

In a chronic MPTP model in Balb/c mice, resveratrol was observed to show significant neuroprotection by alleviating MPTP-induced impairments in motor coordination, oxidative stress, and loss of TH neurons [56]. Furthermore, resveratrol at (10 mg/kg, daily) significantly attenuated toxicity induced by paraquat and maneb, by increasing the levels of cytochrome P450 2D6 gene, as well as the expressions of vesicular monoamine transporter type 2 (VMAT-2). Resveratrol also relieves the increased accumulation of paraquat in nigrostriatal tissues, as well as relieving oxidative stress, microglial activation, neuroinflammation, and increasing the number of TH-positive cells and DA content [57]. Daily oral doses of resveratrol (10, 20 and 40 mg/kg) to rats with 6-OHDA-induced degeneration of the nigrostriatal network revealed that resveratrol alleviates 6-OHDA-induced swelling of mitochondria, condensation of chromatin, and vacuolization of dopaminergic neurons in rat SN. Moreover, resveratrol treatment significantly decreases the m-RNA levels of COX-2 and TNF-*α* in the SN [58]. Other reports also support the neuroprotective effects of resveratrol on nigral cells, wherein it mitigated oxidative damage and depletion of DA in 6-OHDA-induced dopaminergic cell death in a rat model [59–61]. These findings support the role of these natural polyphenols in preventive and/or complementary therapies for several human neurodegenerative diseases caused by oxidative stress and apoptosis.

### 2.6. Acteoside and Echinacoside

Cistanches Herba is the dried juicy stem of *Cistanche deserticola* or *Cistanche tubulosa *(Orobanchaceae) [62]. Total glycosides obtained from Cistanches Herba have been demonstrated to have neuroprotective effects on dopaminergic neurons of SN in a chronically intoxicated MPTP mice model of PD [62]. Treatment with 400 mg/kg of total glycosides significantly improves the altered neurobehavioral pattern of MPTP-intoxicated mice and inhibits the reduction of nigral dopaminergic neurons and the expression of TH in the striatum [62]. Acteoside (Figure 2(b)) extracted from Cistanches Herba has neuroprotective effects against rotenone-induced damage to SH-SY5Y cells. Pretreatment of SH-SY5Y cells with acteoside (10, 20, or 40 mg/L) for 6 h significantly reduces the release of lactate dehydrogenase induced by rotenone (0.5 *μ*M/L). Pretreatment of SH-SY5Y cells with acteoside at the same dose ranges for 6 h, dose dependently decreases the cleavage of parkin induced by 0.5 *μ*M/L of rotenone, decreases *α*-syn-positive SH-SY5Y cells, and stops the dimerization of *α*-syn. These findings indicate that the neuroprotective effects elicited by pretreatment of acteoside are due to its ability to reduce the cleavage of parkin and inhibit the expression of *α*-syn induced by rotenone in SH-SY5Y cells [63]. It is also found that pretreatment with acteoside significantly attenuates LPS-induced release of NO in RAW 264.7 cells via inhibition of NF-*κ*B and activator protein-1 (AP-1) [64]. Acteoside has also been studied for its neuroprotective effects in MPTP models of PD. Pretreatment with acteoside at 10 and 30 mg/kg significantly improved MPTP-induced behavioral deficits in C57BL/6 mice. Acteoside also increases the dopaminergic neurons and content of DA [65]. Echinacoside (Figure 2(c)) is an important bioactive compound obtained and purified from the stems of *Cistanche tubulosa*, a Chinese herbal medicine [66]. Simultaneous treatment with 3.5 and 7.0 mg/kg of echinacoside is observed to prevent the 6-OHDA-induced extracellular loss of monoamine neurotransmitters, including DA, 3,4-dihydroxyphenylacetic acid (DOPAC), and homovanillic acid (HVA), in rat striatum [67]. Further authors suggested that alleviation of MPTP-induced behavioral deficits in C57BL/6 mice by pretreatment of echinacoside might result from a decrease in the biliverdin reductase B level [68]. In this study, acteoside selectively suppressed AP-1 activation, which may be essential for iNOS induction in the LPS-treated macrophages. In another study, prior treatment with echinacoside to MPTP-intoxicated mice was found to increase levels of striatal DA and its metabolite, reduce behavioral deficits, cell death, and lead to a significant rise in TH expression as compared to mice treated with MPTP alone. In addition, pretreatment with echinacoside markedly reduces MPP^+^-induced activations of caspase-3 and caspase-8 in cerebellar granule neurons. These findings indicate that echinacoside uplifts neurochemical and behavioral outcomes in MPTP mice models of PD and inhibits caspase-3 and caspase-8 activation in cerebellar granule neurons [69]. In a comparable study, oral administration of echinacoside (30 mg/kg/day for 14 days) to MPTP-induced sub-acute mice model of PD significantly overcomes the reduction of striatal fibers, nigral dopaminergic neurons, dopamine transporter, and dopamine in MPTP-lesioned animals. As compared to vehicle-treated mice, echinacoside treatment increases mRNA and protein expression of glial cell-derived neurotrophic factor (GDNF) and brain-derived neurotrophic factor in MPTP-lesioned mice. In addition, echinacoside treatment decreases the increased apoptotic cells and mRNA/protein ratio of Bax/Bcl-2 in MPTP-lesioned mice. Echinacoside treatment was also found to improve motor deficits produced by MPTP. These findings demonstrate that echinacoside is an orally active inhibitor of apoptosis, as well as an inducer of neurotrophic factors, and thus providing preclinical support for its therapeutic potential in the treatment of PD [70].

### 2.7. Paeoniflorin

*Paeoniae alba* Radix is the red root of *Paeonia lactiflora* or *Paeonia veitchii*, used extensively as a component of traditional Chinese prescriptions to treat amenorrhea, traumatic injuries, epistaxis, inflammation, boils, and sores and to relieve pain in the chest and costal regions [71]. Paeoniflorin (PF) (Figure 2(d)) is a main principal bioactive component of *P. alba Radix* [72]. PF has been cited to exhibit many pharmacological effects, such as antiinflammatory and antiallergic effects, anti-hyperglycemic effects, analgesic effects, neuromuscular blocking effect, cognition-enhancing effects, and inhibitory effects on steroid protein binding [71]. Pretreatment of PF (2.5 and 5 mg/kg) for 11 days has been shown to protect striatal nerve fibers and TH-positive neurons in SN mitigate bradykinesia observed in an acute MPTP model of PD [71]. Posttreatment with PF for 60 min (2.5 and 5 mg/kg) once a day for the subsequent 3 days after MPTP administration significantly ameliorated the dopaminergic neurodegeneration in a dose-dependent manner [71]. MPTP-induced activation of microglia and astrocytes, accompanied with the upregulation of proinflammatory genes, is also significantly attenuated by posttreatment with PF. Further mechanistic studies revealed that the neuroprotective and antineuroinflammatory effects of PF are associated with the activation of adenosine A_1_ receptor [71]. The effect of PF was also studied in neurological impairments following 6-OHDA-induced unilateral striatal lesion in Sprague-Dawley (SD) rat. Subchronic treatment with PF (2.5, 5 and 10 mg/kg, subcutaneously, twice daily for 11 days dose dependently reduces apomorphine-induced rotation, indicating that PF has an alleviative effect on the 6-OHDA-induced neurological impairments). Since PF had no direct action on dopamine D_1_ receptor or dopamine D_2_ receptor, these results suggest that PF might provide an opportunity to develop a nondopaminergic management of PD [73].

In a recent report, PF was observed to protect PC12 cells from MPP^+^ and acid-induced damage via an autophagic pathway. Treatment with 50 *μ*M of PF protects PC12 cells against both MPP^+^ and acid-induced injury, as determined by MTT assay, and decreases the release of lactate dehydrogenase and apoptotic rate. PF also reduces the influx of Ca^2+^ and reduces its cytosolic content. Further mechanistic study found that the neuroprotective effects of PF were closely associated with the upregulation of LC3-II protein, which is specifically associated with autophagic vacuole membranes. In addition to this, PF also inhibits the MPP^+^-induced overexpression of LAMP2a, which is directly correlated with the activity of the chaperone-mediated autophagy pathway [74]. In a similar study by Sun et al., PF was observed to increase the autophagic degradation of *α*-syn by regulating the expression and activity of acid-sensing ion channels and thus eliciting protective effects against its cytotoxicity in PC12 cells [75].

### 2.8. Tenuigenin

**Figure 3:**
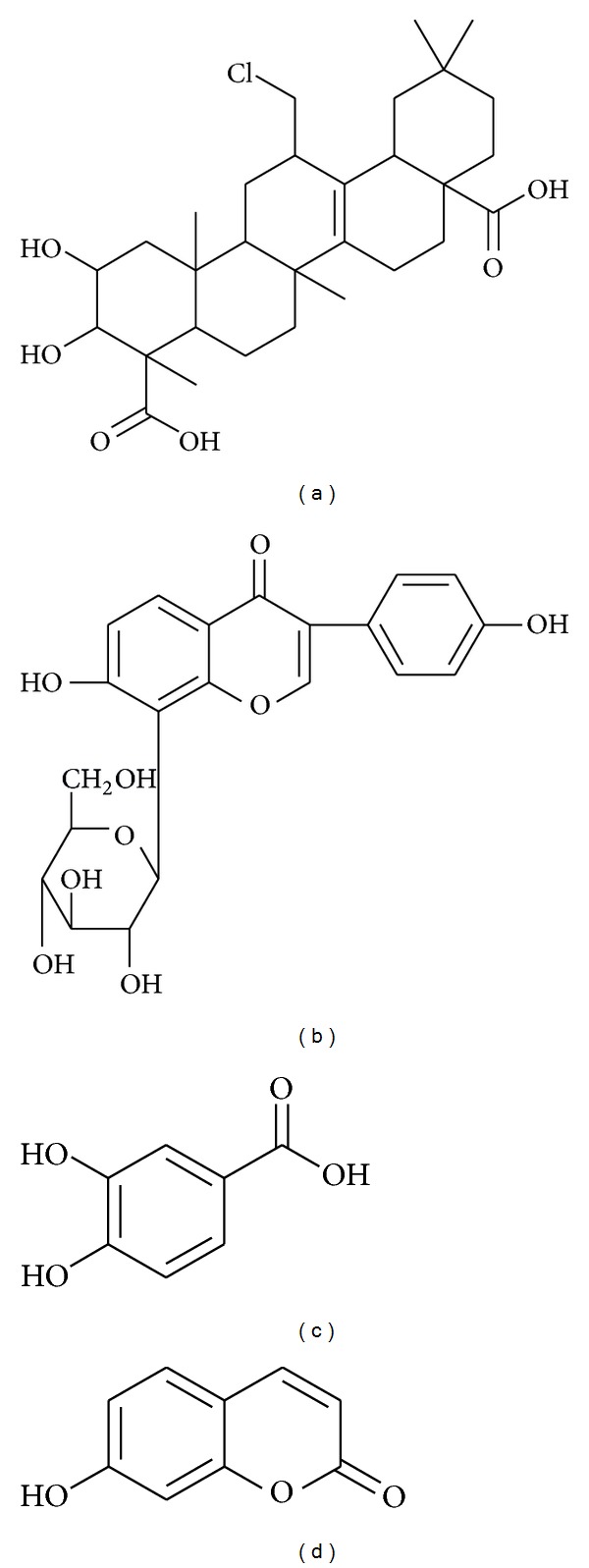
Chemical structure of tenuigenin (a), puerarin (b), protocatechuic acid (c), and umbelliferone (d).

Polygalae radix (PRE) is the dried root of *Polygala tenuifolia* (polygalaceae). PRE is composed of various xanthones, saponins, and oligosaccharide esters [76–78]. PRE is one of the most frequently prescribed herbal remedies in traditional Korean medicine and is used for the treatment of various cognitive symptoms associated with aging, senile dementia, and PD [79, 80]. In a recent finding, PRE (0.05–1 *μ*g/mL) was demonstrated to significantly inhibit 6-OHDA induced damage to PC12 cells, with a maximal effect observed at a dose of 0.1 *μ*g/mL. PRE at 0.1 *μ*g/mL ameliorates the production of ROS, NO, and activity of caspase-3. At the same dose, PRE prevents the abnormal shrinking of dendrites and promotes the survival of mesencephalic dopaminergic neurons from MPP^+^-induced toxicity. In an acute MPTP model of PD, pretreatment with PRE (100 mg/kg/day, 3 days) guards dopaminergic neurons and fibers from MPTP-induced toxicity in striatum and SNpc in C57BL/6 mice [81]. Tenuigenin (Figure 3(a)) is a bioactive principle found in *Polygala tenuifolia* root extracts [82]. In one study by Liang et al., tenuigenin was evaluated for its neuroprotective activity in 6-OHDA-induced cytotoxicity in SH-SY5Y cells. This study found that a 10 *μ*M dose of tenuigenin significantly increased cell viability and reduced cell death [82].

Tenuigenin also protects against 6-OHDA induced damage of the mitochondrial membrane and markedly increases glutathione and superoxide dismutase (SOD) expression. Tenuigenin is observed to downregulate caspase-3 activity at the translational level and also to upregulate the expression of TH in 6-OHDA, damaged SH-SY5Y cells. These results establish that tenuigenin has neuroprotective effects on dopaminergic neurons via its antioxidant and antiapoptotic profile [82]. In a very recent report, tenuigenin was discovered to show the neuroprotective effect on neuroinflammation produced by a single unilateral intranigral dose of LPS (10 *μ*g) in adult male SD rat. The authors observed that treatment with 300 mg/kg/day tenuigenin over 14 weeks improved the survival rate of TH-immunoreactive neurons in the SNpc, as compared to a contralateral side. A single dose (200 or 300 mg/kg/day) of tenuigenin significantly improved levels of DA in the striatum. Furthermore, LPS-induced upregulation of cytokines, such as TNF-*α* and IL-1*β*, was also nullified by tenuigenin administration [83].

### 2.9. Peurarin

Puerarin (Figure 3(b)), also known as daidzein-8-C-glucoside, is a major isoflavonoid derived from the Chinese medical herb *Pueraria lobata* belonging to the family of Leguminosae. In China, this herb has been used as a traditional medicine for treating various diseases including cardiovascular disorders, gynecological disease, osteoporosis, and cognitive dysfunction [84, 85]. In a recent report by Zhu et al., puerarin was observed to upregulate the phosphorylation of Akt in MPP^+^-induced cytotoxicity in SH-SY5Y cells. This effect was further confirmed when puerarin-induced activation of Akt phosphorylation was completely blocked by phosphoinositide 3-kinase (PI3K) inhibitor (LY294002). Treatment with LY294002 also blocked the protective effect elicited by puerarin in MPP^+^-induced toxicity to SH-SY5Y cells. Further mechanistic investigation demonstrated that puerarin inhibits the MPP^+^-induced nuclear translocation of p53, Puma (p53-upregulated mediator of apoptosis), expression of Bax, and caspase-3-dependent programmed cell death. This protection was also abolished by treatment with PI3K/Akt inhibitor [86]. In addition to the involvement of the PI3K/Akt pathway in puerarin mediated neuroprotection to SH-SY5Y cells, puerarin is also reported to prevent the dysfunction of the proteasomal system and thereby avoid the accumulation of ubiquitin-conjugated proteins. Meanwhile, pretreatment of SH-SY5Y cells with puerarin significantly reduces the ratio of bcl-2/bax and caspase-3 activity [87]. In a latest study by Li et al., treatment with puerarin to 6-OHDA-lesioned rats was observed to significantly increase the protein expression of DJ-1 and superoxide dismutase-2 in the SN [88]. In recent report by Zheng et al., puerarin was found to suppress LPS-induced release of iNOS and phosphorylation of MAPKs in N9 cells [89]. Similar* in vitro* effects of puerarin are also evident in 6-OHDA-induced neurotoxicity to PC12 cells, wherein puerarin inhibits the MPP^+^-induced phosphorylation of JNK [90]. These antiapoptotic mechanisms of puerarin were also reflected in 6-OHDA-mediated nigrostriatal damage in rats. Intraperitoneal administration of puerarin 0.12 mg/kg/day for 10 days inhibits the 6-OHDA mediated damage to TH-positive neurons and restores the contents of DA and its metabolites. Furthermore, puerarin also increases the expression level of GDNF in the striatum in rats intoxicated with 6-OHDA [91].

### 2.10. Protocatechuic Acid

Alpiniae Oxyphyllae Fructus (AOE) is the dried and ripe seed of *Alpinia oxyphylla* (Zingiberaceae). Ethanolic extract of AOE prevented and renewed 6-OHDA-induced degeneration of dopaminergic neuron and also attenuated deficits in locomotor activity in a zebrafish model of PD. AOE, by attenuating cellular apoptosis, also increased the viability of 6-OHDA-toxined PC12 cells in a dose-dependent manner. A mechanistic study revealed that AOE protected the dopaminergic neuron from 6-OHDA-induced injury by its antioxidant effect, by inhibition of NO production and iNOS expression in PC12 cells, and by its antiinflammatory action, by downregulation of gene expression of IL-1*β* and TNF-*α*. In addition to this, the PI3K-Akt pathway might also be a part of the neuroprotective mechanism of AOE. Protocatechuic acid (PCA) (Figure 3(c)) is one of the active ingredients obtained from AOE [92]. PCA is found to inhibit the decreased expression of TH, apoptotic morphology, cytotoxicity, mitochondrial dysfunction [93], and abnormal tangling of *α*-syn in PC12 cells treated with MPP^+^ [94]. Neuroprotective activity of PCA is also reported in MPTP-induced neurotoxicity C57BL/6 mice. PCA improved the motor deficits in rotarod test, contents of DA in striatum, and expression of TH in SN of C57BL/6 mice intoxicated by MPTP [95].

## 3. Evidence-Linked Bioactive Components Exhibiting Neuroprotective Activity in *In Vivo* and *In Vitro* Models of PD

**Figure 4:**
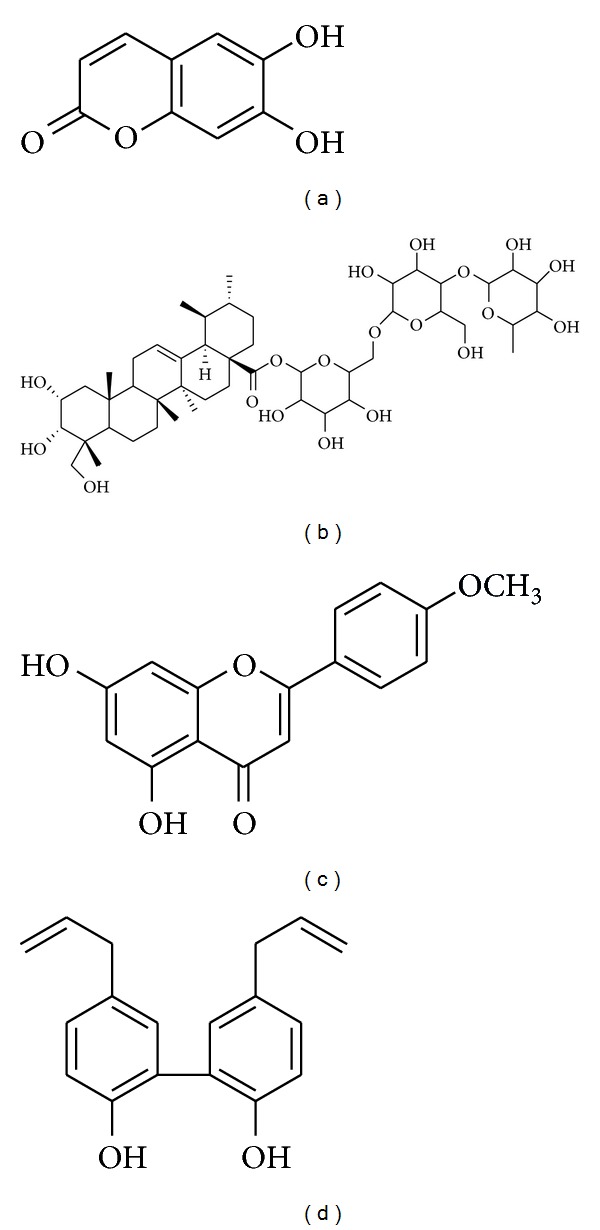
Chemical structure of esculetin (a), asiaticoside (b), acacetin (c), and magnolol (d).

**Figure 5:**
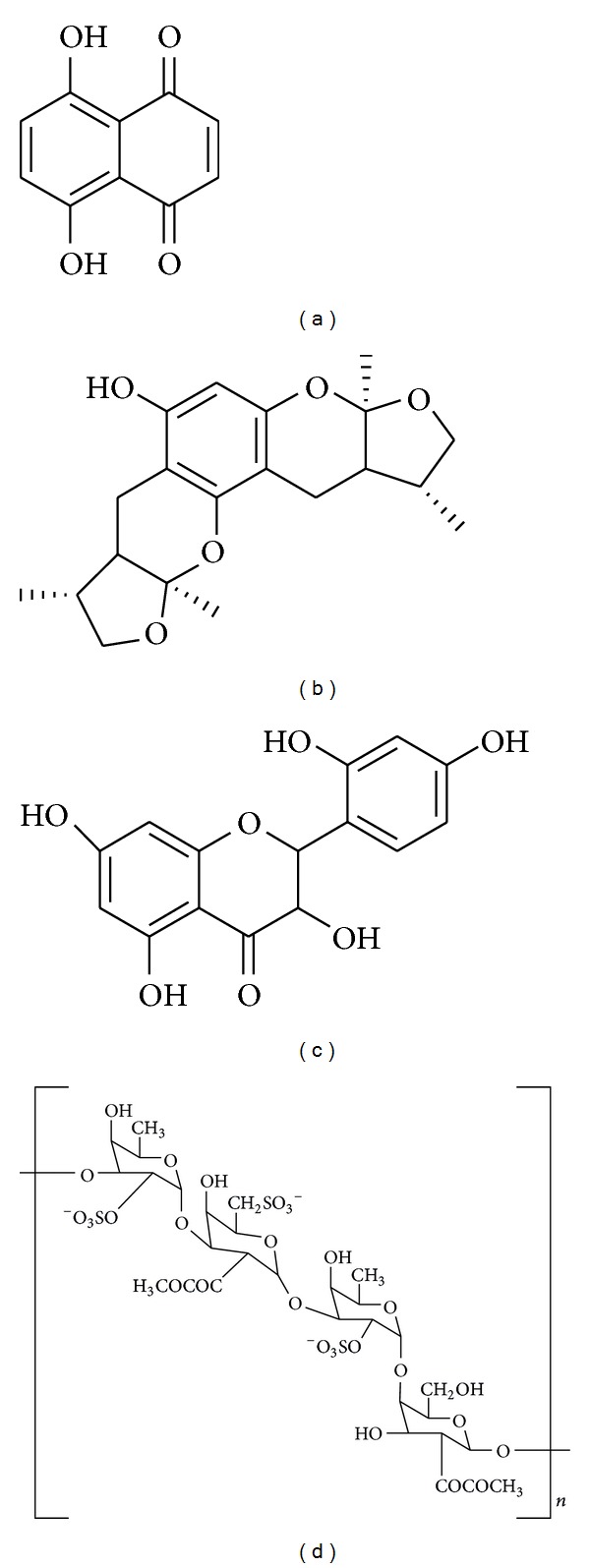
Chemical structure of naphthazarin (a), xyloketal B (b), morin (c), and fucoidan (d).

**Figure 6:**
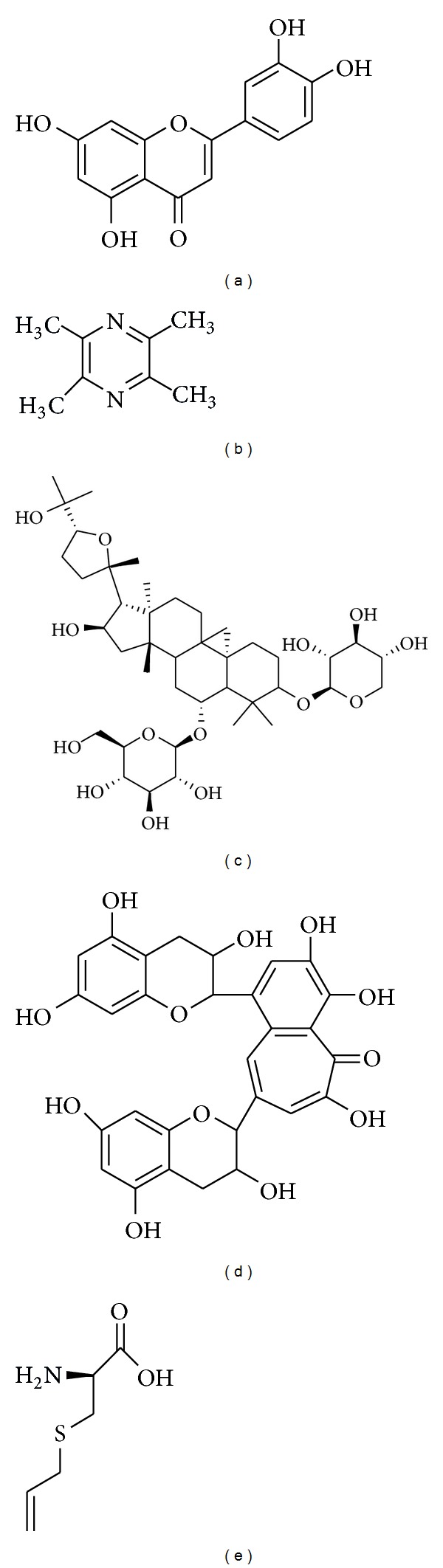
Chemical structure of luteolin (a), tetramethylpyrazine (b), astragaloside IV (c), theaflavin (d), and S-allylcysteine (e).

See Table 1 and Figures 4, 5, and 6.

## 4. Conclusion

PD as a disease has multifactorial pathological mechanisms, and till now currently available conventional treatments are not been able to elicit disease modifying effects by targeting each of these pathomechanisms. Herbal medicines have been known to possess a combination of bioactive components which might target different pathomechanisms in neurodegenerative diseases. Recently, the identification and characterization of medicinal plants to cure PD by conventional medicine is one of the major increasing scientific interest. Although there are more than 120 traditional medicines being used for therapy of central nervous system (CNS) disorders in Asian countries, lack of their quality control data and safety in consumption across the population limits their use in modern world of medicines. A significant amount of people in the developing countries now consume CAM as they are viewed as being innately safer than synthetic chemical compounds. From the ethnobotanical and ethnopharmaceutical resources, many of the bioactive compounds from natural sources have recently been reported to exert neuroprotective effects in various experimental models of PD. Although demand for bioactive compounds from natural sources is increasing, a large-scale, double-blind, and placebo-controlled trials and there pharmacokinetic data to optimize the dosage form are still required to establish the clinical effect of CAM on PD. In addition to this, a very important property of a neuroprotective agent depends on its ability to cross the blood-brain barrier (BBB), in order to reach the target sites of the CNS. Whereas there have been a limited number of animal and cell a based studies focusing on penetration of BBB. Here, we have searched the literature for the most recent available data about bioactive constituents from natural sources that possess neuroprotective activity in various experimental models of PD. Bioactive constituents listed in this current write-up belong to different chemical classes like including, Terpenes (ginsenoside Rg1, tenuigenin, astragaloside IV), flavones (puerarin, luteolin and baicalein, morin), stilbenoids (resveratrol), phenylpropanoid (echinacoside), phenylethyl glycoside (acteoside), coumarin (umbelliferone and esculetin), and catechol (curcumin and protocatechuic acid). The bioactive ingredients discussed have traditionally been used in many countries for different ailments, and thus providing a basis for their validation in comparison with modern day supplements. Even though the range of these studies reported are not vast, all the mentioned bioactive compounds have demonstrated a significant neuroprotective effect in PD models. Hence, bioactive compounds from natural sources can be used as alternative and valuable sources for anti-Parkinsonian drugs.

## Figures and Tables

**Table 1:** 

Bioactive compound	Biological source	Model	Biological effect	References
Umbelliferone (Figure 3(d)) and esculetin (Figure 4(a))	Various plant species	Subacute MPTP model of PD in mice	Decrease in nitrosative stress, protection of tyrosine-hydroxylase- (TH-) positive neurons- and attenuation of caspase-3 activity	[96]

Asiaticoside (Figure 4(b))	*Centella* *Asiatica *	MPTP-induced parkinsonism in rats	Protection of dopaminergic neuron, alleviation of oxidative stress and motor dysfunction	[97]

Acacetin (Figure 4(c))	Chrysanthemum, safflower,* Calamintha* and *Linaria* species	MPP^+^-induced toxicity in primary mesencephalic culture	Protection of dopaminergic neuron and inhibition of production of inflammatory factors	[98, 99]
		Subacute MPTP model of PD in mice	Protection of dopaminergic neuron, avoidance of dopamine (DA) depletion, and alleviation of behavioral deficits	
		Lipopolysaccharide stimulated BV-2 microglial cells	Significant inhibition of NO, prostaglandin E2, iNOS, COX-2, TNF-*α*, and IL-1*β* in LPS stimulated BV-2 cells	

Magnolol (Figure 4(d))	*Magnolia obovata *	MPP^+^-induced toxicity in SH-SY5Y cells	Protection of MPTP-induced cytotoxicity and mitigation of oxidative stress	[100–102]
		Acute MPTP model of PD in mice	Attenuation of MPTP-induced decrease in DAT and TH protein levels and lipid peroxidation in striatum	
		6-OHDA model of PD in mice	Amelioration of apomorphine-induced contralateral rotation and increase of TH protein expression in striatum	
		Lipopolysaccharide + Interferon-*γ*-(IFN-*γ*) stimulated BV-2 and HAPI cells	Inhibition of LPS + IFN-*γ*-induced NO, cytokine, and ROS expression in BV-2 and HAPI cells	

Naphthazarin (Figure 5(a))	*Lomatia obliqua *	Acute MPTP model of PD in mice	Protection of dopaminergic neuron and suppression of astroglial response	[103]

Xyloketal B (Figure 5(b))	Xylaria species	MPP^+^-induced neurotoxicity in Caenorhabditis elegans (C. elegans) and PC12 cells	Increases cell viability in C. elegans and PC12 cells, attenuation of intracellular ROS accumulation, and restoration of GSH level in PC12 cells	[104]

Morin (Figure 5(c))	Onion, red wine and Osage orange	MPP^+^-induced toxicity in PC12 cells	Attenuation of cell viability, reactive oxygen species (ROS), and apoptosis in PC12 cells	[105]
		Subacute MPTP model of PD in mice	Attenuation of behavioral deficits, dopaminergic neuronal death, and striatal dopamine depletion	

Fucoidan (Figure 5(d))	*Laminaria japonica *	MPP^+^-induced toxicity in MN9D cells	Protection of MN9D cells	[106, 107]
		Acute MPTP model of PD in mice	Reduced behavioral deficits, oxidative stress and cell death, increase in striatal dopamine and TH expression	
		LPS-induced damage to rat neurons and primary microglia	Downregulation of intracellular ROS and cytokines release in LPS-activated microglia	

Luteolin (Figure 6(a))	Celery, perilla leaf and chamomile tea.	LPS-induced cell toxicity in primary mesencephalic neuron-glia cultures	Inhibition of LPS-induced activation of microglia and excessive production of TNF-*α*, NO, and superoxide	[108]

Tetramethylpyrazine (Figure 6(b))	*Ligusticum chuanxiong *	MPP^+^-induced toxicity to rat mesencephalic neurons	Increase of dopaminergic neurons and its neurite length	[109, 110]
		Subacute MPTP model of PD in mice	Increase in density of dopaminergic neurons	
		LPS-stimulated N9 microglial cells	Inhibition of NO and iNOS through blocking MAPK and PI3K/Akt activation and reducing ROS production	

Astragaloside IV (Figure 6(c))	*Astragalus membranaceus *	MPP^+^-induced toxicity in SH-SY5Y cells	Reduction in cell loss, activity of caspase-3, ROS, and increase in Bax/Bcl-2 ratio	[111, 112]
		6-OHDA-induced toxicity in primary nigral culture	Promotion of neurite outgrowth and increase in TH-positive neurons	

Theaflavin (Figure 6(d))	*Camellia sinensis *	Subacute MPTP model of PD in mice	Reduction in oxidative stress, motor deficits, and increase in the expression of dopamine transporter (DAT) and VMAT2 in striatum and SN	[113, 114]
		Chronic MPTP/probenecid model of PD in mice	Attenuation of caspase-3, 8, 9 expression, increase in nigral TH and DAT	

S-Allylcysteine (Figure 6(e))	*Allium sativum *	Subacute MPTP model of PD in mice	Reduction in TNF-*α*, inducible iNOS, and glial fibrillary acidic protein (GFAP) expression	[115, 116]
		MPP^+^-induced striatal damage in mice	Attenuation of MPP^+^-induced loss of striatal DA level, oxidative stress, and behavioral deficits	

## References

[1] Liu S-M, Li X-Z, Huo Y, Lu F (2012). Protective effect of extract of Acanthopanax senticosus harms on dopaminergic neurons in Parkinson’s disease mice. *Phytomedicine*.

[2] Schapira AHV, Bezard E, Brotchie J (2006). Novel pharmacological targets for the treatment of Parkinson’s disease. *Nature Reviews Drug Discovery*.

[3] Singh S, Dikshit M (2007). Apoptotic neuronal death in Parkinson’s disease: involvement of nitric oxide. *Brain Research Reviews*.

[4] More SV, Kumar H, Kim IS, Koppulla S, Kim BW, Choi DK (2013). Strategic selection of neuroinflammatory models in Parkinson's disease: evidence from experimental studies. *CNS & Neurological Disorders Drug Targets*.

[5] Cannon JR, Greenamyre JT (2010). Neurotoxic in vivo models of Parkinson’s disease. Recent advances. *Progress in Brain Research*.

[6] Fox SH, Brotchie JM (2010). The MPTP-lesioned non-human primate models of Parkinson’s disease. Past, present, and future. *Progress in Brain Research*.

[7] Manyam BV, Sánchez-Ramos JR (1999). Traditional and complementary therapies in Parkinson’s disease. *Advances in Neurology*.

[8] Manyam BV (1990). Paralysis agitans and levodopa in ’Ayurveda’: ancient Indian medical treatise. *Movement Disorders*.

[9] Lieu CA, Kunselman AR, Manyam BV, Venkiteswaran K, Subramanian T (2010). A water extract of *Mucuna pruriens* provides long-term amelioration of parkinsonism with reduced risk for dyskinesias. *Parkinsonism and Related Disorders*.

[10] Manyam BV, Dhanasekaran M, Hare TA (2004). Neuroprotective effects of the antiparkinson drug *Mucuna pruriens*. *Phytotherapy Research*.

[11] Katzenshlager R, Evans A, Manson A (2004). *Mucuna pruriens* in Parkinson’s disease: a double blind clinical and pharmacological study. *Journal of Neurology, Neurosurgery and Psychiatry*.

[12] Song J-X, Sze SC-W, Ng T-B (2012). Anti-Parkinsonian drug discovery from herbal medicines: what have we got from neurotoxic models?. *Journal of Ethnopharmacology*.

[13] Li XZ, Zhang SN, Liu SM, Lu F (2013). Recent advances in herbal medicines treating Parkinson's disease. *Fitoterapia*.

[14] Chung V, Liu L, Bian Z (2006). Efficacy and safety of herbal medicines for idiopathic Parkinson’s disease: a systematic review. *Movement Disorders*.

[15] Kumar GP, Khanum F (2012). Neuroprotective potential of phytochemicals. *Pharmacognosy Reviews*.

[16] Lobo V, Patil A, Phatak A, Chandra N (2010). Free radicals, antioxidants and functional foods: impact on human health. *Pharmacognosy Reviews*.

[17] Li Q, Zhao D, Bezard E (2006). Traditional Chinese medicine for Parkinson's disease: a review of Chinese literature. *Behavioural Pharmacology*.

[18] Ip PS, Tsim KW, Chan K, Bauer R (2012). Application of complementary and alternative medicine on neurodegenerative disorders: current status and future prospects. *Evidence-Based Complementary and Alternative Medicine*.

[19] Saklani A, Kutty SK (2008). Plant-derived compounds in clinical trials. *Drug Discovery Today*.

[20] Hu S, Han R, Mak S, Han Y (2011). Protection against 1-methyl-4-phenylpyridinium ion (MPP^+^)-induced apoptosis by water extract of ginseng (Panax ginseng C.A. Meyer) in SH-SY5Y cells. *Journal of Ethnopharmacology*.

[21] Radad K, Gille G, Moldzio R, Saito H, Rausch W-D (2004). Ginsenosides Rb1 and Rg1 effects on mesencephalic dopaminergic cells stressed with glutamate. *Brain Research*.

[22] Luo F-C, Wang S-D, Li K, Nakamura H, Yodoi J, Bai J (2010). Panaxatriol saponins extracted from Panax notoginseng induces thioredoxin-1 and prevents 1-methyl-4-phenylpyridinium ion-induced neurotoxicity. *Journal of Ethnopharmacology*.

[23] Luo F-C, Wang S-D, Qi L, Song J-Y, Lv T, Bai J (2011). Protective effect of panaxatriol saponins extracted from Panax notoginseng against MPTP-induced neurotoxicity in vivo. *Journal of Ethnopharmacology*.

[24] Liu Q, Kou J-P, Yu B-Y (2011). Ginsenoside Rg1 protects against hydrogen peroxide-induced cell death in PC12 cells via inhibiting NF-*κ*B activation. *Neurochemistry International*.

[25] Xu H, Jiang H, Wang J, Xie J (2010). Rg1 protects iron-induced neurotoxicity through antioxidant and iron regulatory proteins in 6-OHDA-treated MES23.5 cells. *Journal of Cellular Biochemistry*.

[26] Xu H, Jiang H, Wang J, Xie J (2010). Rg1 protects the MPP^+^-treated MES23.5 cells via attenuating DMT1 up-regulation and cellular iron uptake. *Neuropharmacology*.

[27] Wang J, Xu H-M, Yang H-D, Du X-X, Jiang H, Xie J-X (2009). Rg1 reduces nigral iron levels of MPTP-treated C57BL6 mice by regulating certain iron transport proteins. *Neurochemistry International*.

[28] Hu J-F, Song X-Y, Chu S-F (2011). Inhibitory effect of ginsenoside Rg1 on lipopolysaccharide-induced microglial activation in mice. *Brain Research*.

[29] Xu L, Liu LX, Chen WF, Xie JX, Huang WX (2008). The protective effect of ginsenoside Rg1 on dopaminergic neurons of substantia in the ovariectomized rat model of Parkinson's disease. *Zhongguo Ying Yong Sheng Li Xue Za Zhi*.

[30] Xu L, Chen W-F, Wong M-S (2009). Ginsenoside Rg1 protects dopaminergic neurons in a rat model of Parkinson’s disease through the IGF-I receptor signalling pathway. *British Journal of Pharmacology*.

[31] Jeong K, Shin Y-C, Park S (2011). Ethanol extract of Scutellaria baicalensis Georgi prevents oxidative damage and neuroinflammation and memorial impairments in artificial senescense mice. *Journal of Biomedical Science*.

[32] Li X-X, He G-R, Mu X (2012). Protective effects of baicalein against rotenone-induced neurotoxicity in PC12 cells and isolated rat brain mitochondria. *European Journal of Pharmacology*.

[33] Mu X, He G, Cheng Y, Li X, Xu B, Du G (2009). Baicalein exerts neuroprotective effects in 6-hydroxydopamine-induced experimental parkinsonism in vivo and in vitro. *Pharmacology Biochemistry and Behavior*.

[34] Yu X, He GR, Sun L (2012). Assessment of the treatment effect of baicalein on a model of Parkinsonian tremor and elucidation of the mechanism. *Life Sciences*.

[35] Mu X, He G-R, Yuan X, Li X-X, Du G-H (2011). Baicalein protects the brain against neuron impairments induced by MPTP in C57BL/6 mice. *Pharmacology Biochemistry and Behavior*.

[36] Cheng Y, He G, Mu X (2008). Neuroprotective effect of baicalein against MPTP neurotoxicity: behavioral, biochemical and immunohistochemical profile. *Neuroscience Letters*.

[37] Jiang M, Porat-Shliom Y, Pei Z (2010). Baicalein reduces E46K *α*-synuclein aggregation in vitro and protects cells against E46K *α*-synuclein toxicity in cell models of familiar Parkinsonism. *Journal of Neurochemistry*.

[38] Lu J-H, Ardah MT, Durairajan SSK (2011). Baicalein inhibits formation of alpha-synuclein oligomers within living cells and prevents Abeta peptide fibrillation and oligomerisation. *ChemBioChem*.

[39] Marchiani A, Rozzo C, Fadda A, Delogu G, Ruzza P Curcumin and curcumin-like molecules: from spice to drugs.

[40] Gupta SC, Patchva S, Koh W, Aggarwal BB (2012). Discovery of curcumin, a component of golden spice, and its miraculous biological activities. *Clinical and Experimental Pharmacology and Physiology*.

[41] Jiang TF, Zhang YJ, Zhou HY (2013). Curcumin ameliorates the neurodegenerative pathology in A53T alpha-synuclein cell model of Parkinson's disease through the downregulation of mTOR/p70S6K signaling and the recovery of macroautophagy. *Journal of NeuroImmune Pharmacology*.

[42] Wang MS, Boddapati S, Emadi S, Sierks MR (2010). Curcumin reduces *α*-synuclein induced cytotoxicity in Parkinson’s disease cell model. *BMC Neuroscience*.

[43] Ojha RP, Rastogi M, Devi BP, Agrawal A, Dubey GP (2012). Neuroprotective effect of curcuminoids against inflammation-mediated dopaminergic neurodegeneration in the MPTP model of Parkinson's disease. *Journal of Neuroimmune Pharmacology*.

[44] Pan J, Li H, Ma JF (2012). Curcumin inhibition of JNKs prevents dopaminergic neuronal loss in a mouse model of Parkinson's disease through suppressing mitochondria dysfunction. *Translational Neurodegeneration*.

[45] Tripanichkul W, Jaroensuppaperch E-O (2012). Curcumin protects nigrostriatal dopaminergic neurons and reduces glial activation in 6-hydroxydopamine hemiparkinsonian mice model. *International Journal of Neuroscience*.

[46] An H, Kim IS, Koppula S (2010). Protective effects of Gastrodia elata Blume on MPP^+^-induced cytotoxicity in human dopaminergic SH-SY5Y cells. *Journal of Ethnopharmacology*.

[47] Kim IS, Choi D-K, Jung HJ (2011). Neuroprotective effects of vanillyl alcohol in gastrodia elata blume through suppression of oxidative stress and anti-apoptotic activity in toxin-induced dopaminergic MN9D cells. *Molecules*.

[48] Kumar H, Kim IS, More SV, Kim BW, Bahk YY, Choi DK (2013). Gastrodin protects apoptotic dopaminergic neurons in a toxin-induced Parkinson's disease model. *Evidence-Based Complementary and Alternative Medicine*.

[49] Dai J-N, Zong Y, Zhong L-M (2011). Gastrodin inhibits expression of inducible no synthase, cyclooxygenase-2 and proinflammatory cytokines in cultured LPS-Stimulated microglia via MAPK pathways. *PLoS ONE*.

[50] Frémont L (2000). Biological effects of resveratrol. *Life Sciences*.

[51] Bhat KPL, Kosmeder JW, Pezzuto JM (2001). Biological effects of resveratrol. *Antioxidants and Redox Signaling*.

[52] Chang CY, Choi DK, Lee DK, Hong YJ, Park EJ (2013). Resveratrol confers protection against rotenone-induced neurotoxicity by modulating myeloperoxidase levels in glial cells. *PLoS ONE*.

[53] Wu Y, Li X, Zhu JX (2011). Resveratrol-activated AMPK/SIRT1/autophagy in cellular models of Parkinson’s disease. *NeuroSignals*.

[54] Bournival J, Quessy P, Martinoli M-G (2009). Protective effects of resveratrol and quercetin against MPP^+^-induced oxidative stress act by modulating markers of apoptotic death in dopaminergic neurons. *Cellular and Molecular Neurobiology*.

[55] Alvira D, Yeste-Velasco M, Folch J (2007). Comparative analysis of the effects of resveratrol in two apoptotic models: inhibition of complex I and potassium deprivation in cerebellar neurons. *Neuroscience*.

[56] Lu K-T, Ko M-C, Chen B-Y (2008). Neuroprotective effects of resveratrol on MPTP-induced neuron loss mediated by free radical scavenging. *Journal of Agricultural and Food Chemistry*.

[57] Srivastava G, Dixit A, Yadav S, Patel DK, Prakash O, Singh MP (2012). Resveratrol potentiates cytochrome P450 2 d22-mediated neuroprotection in maneb- and paraquat-induced parkinsonism in the mouse. *Free Radical Biology and Medicine*.

[58] Jin F, Wu Q, Lu Y-F, Gong Q-H, Shi J-S (2008). Neuroprotective effect of resveratrol on 6-OHDA-induced Parkinson’s disease in rats. *European Journal of Pharmacology*.

[59] Khan MM, Ahmad A, Ishrat T (2010). Resveratrol attenuates 6-hydroxydopamine-induced oxidative damage and dopamine depletion in rat model of Parkinson’s disease. *Brain Research*.

[60] Wang Y, Xu H, Fu Q, Ma R, Xiang J (2011). Protective effect of resveratrol derived from Polygonum cuspidatum and its liposomal form on nigral cells in Parkinsonian rats. *Journal of the Neurological Sciences*.

[61] Chao J, Li H, Cheng K-W, Yu M-S, Chang RC-C, Wang M (2010). Protective effects of pinostilbene, a resveratrol methylated derivative, against 6-hydroxydopamine-induced neurotoxicity in SH-SY5Y cells. *Journal of Nutritional Biochemistry*.

[62] Li W-W, Yang R, Cai D-F (2008). Protective effects of Cistanche total glycosides on dopaminergic neuron in substantia nigra of model mice of Parkinson’s disease. *Chinese Journal of Integrated Traditional and Western Medicine*.

[63] Gao Y, Pu X-P (2007). Neuroprotective effect of acteoside against rotenone-induced damage of SH-SY5Y cells and its possible mechanism. *Chinese Pharmacological Bulletin*.

[64] Lee JY, Woo E-R, Kang KW (2005). Inhibition of lipopolysaccharide-inducible nitric oxide synthase expression by acteoside through blocking of AP-1 activation. *Journal of Ethnopharmacology*.

[65] Zhao L, Pu X-P (2007). Neuroprotective effect of acteoside against MPTP-induced mouse model of Parkinsons disease. *Chinese Pharmacological Bulletin*.

[66] Xie J, Deng J, Tan F, Su J (2010). Separation and purification of echinacoside from *Penstemon barbatus* (Can.) Roth by recycling high-speed counter-current chromatography. *Journal of Chromatography B*.

[67] Chen H, Jing FC, Li CL, Tu PF, Zheng QS, Wang ZH (2007). Echinacoside prevents the striatal extracellular levels of monoamine neurotransmitters from diminution in 6-hydroxydopamine lesion rats. *Journal of Ethnopharmacology*.

[68] Zhao X, Pu X-P, Geng X-C (2008). Effects of echinacoside on protein expression from substantia nigra and striatal tissue in mouse MPTP model of Parkinsons disease by using 2-dimensional electrophoresis analysis. *Chinese Pharmacological Bulletin*.

[69] Geng X, Tian X, Tu P, Pu X (2007). Neuroprotective effects of echinacoside in the mouse MPTP model of Parkinson’s disease. *European Journal of Pharmacology*.

[70] Zhao Q, Gao J, Li W, Cai D (2010). Neurotrophic and neurorescue effects of Echinacoside in the subacute MPTP mouse model of Parkinson’s disease. *Brain Research*.

[71] Liu H-Q, Zhang W-Y, Luo X-T, Ye Y, Zhu X-Z (2006). Paeoniflorin attenuates neuroinflammation and dopaminergic neurodegeneration in the MPTP model of Parkinson’s disease by activation of adenosine A1 receptor. *British Journal of Pharmacology*.

[72] Liu D-Z, Xie K-Q, Ji X-Q, Ye Y, Jiang C-L, Zhu X-Z (2005). Neuroprotective effect of paeoniflorin on cerebral ischemic rat by activating adenosine A1 receptor in a manner different from its classical agonists. *British Journal of Pharmacology*.

[73] Liu D-Z, Zhu J, Jin D-Z (2007). Behavioral recovery following sub-chronic paeoniflorin administration in the striatal 6-OHDA lesion rodent model of Parkinson’s disease. *Journal of Ethnopharmacology*.

[74] Cao B-Y, Yang Y-P, Luo W-F (2010). Paeoniflorin, a potent natural compound, protects PC12 cells from MPP^+^ and acidic damage via autophagic pathway. *Journal of Ethnopharmacology*.

[75] Sun X, Cao Y-B, Hu L-F (2011). ASICs mediate the modulatory effect by paeoniflorin on alpha-synuclein autophagic degradation. *Brain Research*.

[76] Jiang Y, Tu P-F (2002). Xanthone O-glycosides from *Polygala tenuifolia*. *Phytochemistry*.

[77] Jiang Y, Tu P (2005). Four new phenones from the cortexes of *Polygala tenuifolia*. *Chemical and Pharmaceutical Bulletin*.

[78] Jiang Y, Zhang W, Tu P, Xu X (2005). Xanthone glycosides from *Polygala tenuifolia* and their conformational analyses. *Journal of Natural Products*.

[79] Yan M (2006). Studies on antiaging action of *Polygala tenuifolia* Wild. *Journal of Clinical Pharmacy and Therapeutics*.

[80] Zhang H, Han T, Zhang L (2008). Effects of tenuifolin extracted from radix polygalae on learning and memory: a behavioral and biochemical study on aged and amnesic mice. *Phytomedicine*.

[81] Choi JG, Kim HG, Kim MC (2011). Polygalae radix inhibits toxin-induced neuronal death in the Parkinson’s disease models. *Journal of Ethnopharmacology*.

[82] Liang Z, Shi F, Wang Y (2011). Neuroprotective effects of tenuigenin in a SH-SY5Y cell model with 6-OHDA-induced injury. *Neuroscience Letters*.

[83] Yuan HL, Li B, Xu J (2012). Tenuigenin protects dopaminergic neurons from inflammation-mediated damage induced by the lipopolysaccharide. *CNS Neuroscience & Therapeutics*.

[84] Chang Y, Hsieh C-Y, Peng Z-A (2009). Neuroprotective mechanisms of puerarin in middle cerebral artery occlusion-induced brain infarction in rats. *Journal of Biomedical Science*.

[85] Han R-M, Tian Y-X, Becker EM, Andersen ML, Zhang J-P, Skibsted LH (2007). Puerarin and conjugate bases as radical scavengers and antioxidants: molecular mechanism and synergism with *β*-carotene. *Journal of Agricultural and Food Chemistry*.

[86] Zhu G, Wang X, Wu S, Li Q (2012). Involvement of activation of PI3K/Akt pathway in the protective effects of puerarin against MPP^+^-induced human neuroblastoma SH-SY5Y cell death. *Neurochemistry International*.

[87] Cheng Y-F, Zhu G-Q, Wang M (2009). Involvement of ubiquitin proteasome system in protective mechanisms of Puerarin to MPP^+^-elicited apoptosis. *Neuroscience Research*.

[88] Li R, Zheng N, Liang T, He Q, Xu L (2013). Puerarin attenuates neuronal degeneration and blocks oxidative stress to elicit a neuroprotective effect on substantia nigra injury in 6-OHDA-lesioned rats. *Brain Research*.

[89] Zheng G-M, Yu C, Yang Z (2012). Puerarin suppresses production of nitric oxide and inducible nitric oxide synthase in lipopolysaccharide-induced N9 microglial cells through regulating MAPK phosphorylation, O-GlcNAcylation and NF-*κ*B translocation. *International Journal of Oncology*.

[90] Wang G, Zhou L, Zhang Y (2011). Implication of the c-Jun-NH2-terminal kinase pathway in the neuroprotective effect of puerarin against 1-methyl-4-phenylpyridinium (MPP^+^)-induced apoptosis in PC-12 cells. *Neuroscience Letters*.

[91] Zhu G, Wang X, Chen Y (2010). Puerarin protects dopaminergic neurons against 6-hydroxydopamine neurotoxicity via inhibiting apoptosis and upregulating glial cell line-derived neurotrophic factor in a rat model of Parkinson’s disease. *Planta Medica*.

[92] Zhang Z-J, Cheang LCV, Wang M-W (2012). Ethanolic extract of fructus alpinia oxyphylla protects against 6-hydroxydopamine-induced damage of PC12 cells in vitro and dopaminergic neurons in zebrafish. *Cellular and Molecular Neurobiology*.

[93] Guan S, Jiang B, Bao YM, An LJ (2006). Protocatechuic acid suppresses MPP^+^-induced mitochondrial dysfunction and apoptotic cell death in PC12 cells. *Food and Chemical Toxicology*.

[94] Zhang H-N, An C-N, Xu M, Guo D-A, Li M, Pu X-P (2009). Protocatechuic acid inhibits rat pheochromocytoma cell damage induced by a dopaminergic neurotoxin. *Biological and Pharmaceutical Bulletin*.

[95] Zhang H-N, An C-N, Zhang H-N, Pu X-P (2010). Protocatechuic acid inhibits neurotoxicity induced by MPTP in vivo. *Neuroscience Letters*.

[96] Subramaniam SR, Ellis EM (2013). Neuroprotective effects of umbelliferone and esculetin in a mouse model of Parkinson's disease. *Journal of Neuroscience Research*.

[97] Xu C-L, Wang Q-Z, Sun L-M (2012). Asiaticoside: attenuation of neurotoxicity induced by MPTP in a rat model of Parkinsonism via maintaining redox balance and up-regulating the ratio of Bcl-2/Bax. *Pharmacology Biochemistry and Behavior*.

[98] Kim HG, Ju MS, Ha SK, Lee H, Kim SY, Oh MS (2012). Acacetin protects dopaminergic cells against 1-methyl-4-phenyl-1, 2, 3, 6-tetrahydropyridine-induced neuroinflammation in vitro and in vivo. *Biological & Pharmaceutical Bulletin*.

[99] Ha SK, Moon E, Lee P, Ryu JH, Oh MS, Kim SY (2012). Acacetin attenuates neuroinflammation via regulation the response to LPS stimuli in vitro and in vivo. *Neurochemical Research*.

[100] Muroyama A, Fujita A, Lv C, Kobayashi S, Fukuyama Y, Mitsumoto Y (2012). Magnolol protects against MPTP/MPP^+^-Induced toxicity via Inhibition of oxidative stress in In vivo and In vitro models of parkinson's disease. *Parkinson's Disease*.

[101] Chen H-H, Lin S-C, Chan M-H (2011). Protective and restorative effects of magnolol on neurotoxicity in mice with 6-hydroxydopamine-induced hemiparkinsonism. *Neurodegenerative Diseases*.

[102] Chuang DY, Chan MH, Zong Y (2013). Magnolia polyphenols attenuate oxidative and inflammatory responses in neurons and microglial cells. *Journal of Neuroinflammation*.

[103] Choi SY, Son TG, Park HR (2012). Naphthazarin has a protective effect on the 1-methyl-4-phenyl-1,2,3,4-tetrahydropyridine-induced Parkinson’s disease model. *Journal of Neuroscience Research*.

[104] Lu X-L, Yao X-L, Liu Z (2010). Protective effects of xyloketal B against MPP^+^-induced neurotoxicity in Caenorhabditis elegans and PC12 cells. *Brain Research*.

[105] Zhang Z-T, Cao X-B, Xiong N (2010). Morin exerts neuroprotective actions in Parkinson disease models in vitro and in vivo. *Acta Pharmacologica Sinica*.

[106] Luo D, Zhang Q, Wang H (2009). Fucoidan protects against dopaminergic neuron death in vivo and in vitro. *European Journal of Pharmacology*.

[107] Cui YQ, Jia YJ, Zhang T, Zhang QB, Wang XM (2012). Fucoidan protects against lipopolysaccharide-induced rat neuronal damage and inhibits the production of proinflammatory mediators in primary microglia. *CNS Neuroscience & Therapeutics*.

[108] Chen H-Q, Jin Z-Y, Wang X-J, Xu X-M, Deng L, Zhao J-W (2008). Luteolin protects dopaminergic neurons from inflammation-induced injury through inhibition of microglial activation. *Neuroscience Letters*.

[109] Du J, Shan LC, Zhang GX, Wang YQ (2011). Effect of TMP on dopaminergic neuron injury induced by MPTP in vivo and vitro. *Lishizhen Medicine and Materia Medica Research*.

[110] Liu H-T, Du Y-G, He J-L (2010). Tetramethylpyrazine inhibits production of nitric oxide and inducible nitric oxide synthase in lipopolysaccharide-induced N9 microglial cells through blockade of MAPK and PI3K/Akt signaling pathways, and suppression of intracellular reactive oxygen species. *Journal of Ethnopharmacology*.

[111] Zhang Z-G, Wu L, Wang J-L (2012). Astragaloside IV prevents MPP^+^-induced SH-SY5Y cell death via the inhibition of Bax-mediated pathways and ROS production. *Molecular and Cellular Biochemistry*.

[112] Chan W-S, Durairajan SSK, Lu J-H (2009). Neuroprotective effects of Astragaloside IV in 6-hydroxydopamine-treated primary nigral cell culture. *Neurochemistry International*.

[113] Anandhan A, Janakiraman U, Manivasagam T (2012). Theaflavin ameliorates behavioral deficits, biochemical indices and monoamine transporters expression against subacute 1-methyl-4-phenyl-1, 2, 3, 6-tetrahydropyridine (MPTP)-induced mouse model of Parkinson's disease. *Neuroscience*.

[114] Anandhan A, Tamilselvam K, Radhiga T, Rao S, Essa MM, Manivasagam T (2012). Theaflavin, a black tea polyphenol, protects nigral dopaminergic neurons against chronic MPTP/probenecid induced Parkinson’s disease. *Brain Research*.

[115] García E, Villeda-Hernández J, Pedraza-Chaverrí J, Maldonado PD, Santamaría A (2010). S-allylcysteine reduces the MPTP-induced striatal cell damage via inhibition of pro-inflammatory cytokine tumor necrosis factor-*α* and inducible nitric oxide synthase expressions in mice. *Phytomedicine*.

[116] Rojas P, Serrano-García N, Medina-Campos ON, Pedraza-Chaverri J, Maldonado PD, Ruiz-Sánchez E (2011). S-Allylcysteine, a garlic compound, protects against oxidative stress in 1-methyl-4-phenylpyridinium-induced parkinsonism in mice. *Journal of Nutritional Biochemistry*.

